# The Natural History of Non-operatively Managed Legg–Calvé–Perthes’ Disease

**DOI:** 10.1007/s43465-021-00543-x

**Published:** 2022-01-20

**Authors:** Ramez Ailabouni, Bryn O. Zomar, Bronwyn L. Slobogean, Emily K. Schaeffer, Benjamin Joseph, Kishore Mulpuri

**Affiliations:** 1 Department of Orthopaedic Surgery and Musculoskeletal Medicine, University of Otago, Christchurch, New Zealand; 2 Department of Orthopaedic Surgery, BC Children’s Hospital, 1D66-4480 Oak Street, Vancouver, BC V6H 3V4 Canada; 3 Department of Orthopaedics, University of British Columbia, Vancouver, BC Canada; 4 Department of Neurosurgery, Johns Hopkins University School of Medicine, Baltimore, MD USA; 5 (Emeritus) Paediatric Orthopaedic Service, Kasturba Medical College, Manipal, Karnataka State India

**Keywords:** Legg–Calvé–Perthes’, Perthes disease, Natural history, Pediatric hip, Non-operative treatment

## Abstract

**Background:**

The purpose of this study was to examine the evolution of Legg–Calvé–Perthes’ disease (LCPD) among children from British Columbia (BC), Canada who were treated non-operatively and to compare the results to a previously conducted study in India.

**Methods:**

This was a retrospective review of patients treated non-operatively for LCPD in BC between 1990 and 2006 compared with a cohort from India. Demographic and treatment information were collected from medical records. Radiographs were assigned modified Waldenstrom, Catterall, Salter–Thompson and Herring classifications and intra- and interobserver reliability were assessed. We evaluated epiphyseal extrusion (EE) and metaphyseal width (MW), and assessed radiographs using the Mose and modified Stulberg classifications.

**Results:**

102 hips (90 patients) had radiographs available for evaluation. 95% of the BC cohort presented as Waldenstrom stages I and II, whereas, 90% of the Indian cohort presented as IIIa. Final EE was similar for both groups (BC 26.8%, India 27.3%) and final MW was 119% in both groups. Modified Waldenstrom and Herring classifications had substantial intra- and interobserver reliability, while Salter–Thompson and Catterall classifications had moderate agreement at best. Most hips were Catterall IV (80%) and Herring C (89%) for the BC cohort compared to only 44% and 43% of Indian hips, respectively. Most hips were irregular according to the Mose classification (BC 43%, India 52%) and aspherical according to the Stulberg classification (BC 78%).

**Conclusions:**

We found similar radiographic progression and final radiographic appearances of LCPD in India and BC though differences in the distribution of the classification systems warrant further study.

**Supplementary Information:**

The online version contains supplementary material available at 10.1007/s43465-021-00543-x.

## Introduction

Legg–Calvé–Perthes’ disease (LCPD) is a self-limiting pediatric hip disorder characterized by avascular necrosis (AVN) of the capital femoral epiphysis. The affected femoral epiphysis undergoes necrosis, then progresses through fragmentation, reconstitution and healing over time without treatment. These stages were described by Waldenstrom [1] and modified by Joseph et al. [2]. The incidence of LCPD varies with race, geographic location, and socioeconomic status [3–11]; however, it remains unknown whether there are differences in the evolution of the disease among children from different geographic locations.

The relevance of understanding the disease process and its evolution in different populations is gaining importance due to the globalization of clinical research. In 2004, an estimated 20–30% of all clinical trials were conducted in developing countries [12]. Some of the advantages of recruiting patients from developing nations include large populations, rapid recruitment of subjects, and low overhead costs [13, 14]. Establishing that the natural history of a disease is similar in different parts of the world is key to the generalizability of the results of international research [13, 15]. Furthermore, establishing similarity, or lack thereof, between remote locations may affect the extension of international collaborative groups examining diseases such as LCPD. Consequently, comparisons of the patterns of disease evolution across different geographic locations are warranted.

The purpose of this study was to examine the evolution of LCPD among children from British Columbia, Canada who were treated non-operatively and to compare the results to a similar study previously conducted in south-west India [2]. We investigated the following questions: [1] Is the radiographic evolution of LCPD comparable between British Columbia and south-west India, [2] Is the timing of epiphyseal extrusion and metaphyseal widening comparable between the two populations, and [3] Are the radiographic outcomes of non-operatively treated LCPD similar among children from British Columbia and south-west India?

## Materials and Methods

A retrospective chart review was conducted of patients treated non-operatively for LCPD at a tertiary pediatric hospital in British Columbia, Canada. Results of this review were then compared to those of a similar study performed on a cohort of 610 children from India [2]. Research Ethics Board (REB) approval was obtained for this review. All patients who were managed non-operatively between 1990 until 2006 were included in the study. For the purposes of this study, operative treatment was defined as any bony containment procedure such as femoral or pelvic osteotomies. Non-operative treatments included traction, botulinum toxin (Botox) injections, casting, bracing, crutches, and manipulation, some of which was undertaken under general anesthesia. Exclusion criteria included epiphyseal dysplasia, sickle cell anemia and children with LCPD who were older than 12 years at the onset of symptoms.

The patients’ medical records and x-rays were reviewed and details of the age at onset of symptoms, age at presentation, sex, involved side, and treatment were recorded. Available radiographs (anteroposterior [AP] and Lauenstein frog-lateral [lateral]) were reviewed and assigned a staging classification based on Joseph et al.’s modification of the Waldenstrom system [2, 16] (Online Resource 1). Radiographs were assigned Catterall [17], Salter–Thompson [18] and Herring [19] classifications where appropriate. A total of 100 radiographs from the study population were randomly selected to assess the intra- and interobserver reliability of the modified Waldenstrom, Herring, Salter–Thompson, and Catterall classification systems. The prognostic classification systems were only assigned where possible as they are dependent on having radiographs at specific time points in the disease process. One evaluator classified the radiographs on two separate occasions several days apart to determine the intraobserver reliability. A second blinded reviewer classified the same imaging set on a separate occasion to determine the interobserver reliability.

Epiphyseal extrusion was evaluated by Reimer’s migration percentage [20]. The metaphyseal width was calculated in unilateral cases by measuring the widest point of the metaphysis parallel to the physeal line. This was calculated on both the AP and lateral views, and the greater of the two measures was used. The degree of widening of the metaphysis was expressed as a percentage ratio of the metaphyseal width of the affected femur divided by the normal side. The short-term outcome of the disease was assessed in patients who reached the healed stage (stage IV) using the Mose criteria [20] and the modified Stulberg classification system [21–23]. The only difference in methodology from the series in south-west India was the addition of assigning the modified Stulberg classification [2].

Due to a small sample size, robust statistical analysis of our data was not possible and hence, only descriptive statistics were used in this study. Continuous variables were summarized using means with a 95% confidence interval or medians with interquartile ranges (IQR), as appropriate. Categorical variables were summarized using counts and proportions. The Kappa statistic was computed to test the degree of intra- and interobserver agreement in the assignment of the classification systems and the results were interpreted in the method of Landis and Koch [24].

## Results

One hundred twenty-five consecutive hips with LCPD were treated at our centre from 1990 to 2006. Radiographs were unavailable for 23 hips and were excluded from analysis therefore, the study population comprised 102 hips in 90 patients. A total of 767 pairs of AP and lateral radiographs were available for analysis. Sequential radiographs from presentation to complete healing were available for 62 hips in 58 patients (64%). Of these 62 hips, only five had radiographs classifiable at every modified Waldenstrom stage. Therefore, meaningful calculations of disease duration were not possible as part of our study. Although we did not have complete radiographic sets for all patients from presentation to healing, relevant information about the stages of the disease could still be obtained from the available serial radiographs. The number of radiographs available for review for each patient ranged from two to 19. The median number of days between radiographs was 175 (IQR 91, 357).

Demographics of the children from BC and India are outlined in Table 1. The majority of affected patients were male in both groups. The mean age at presentation in BC was considerably younger than in India for both sexes. The incidence of bilateral disease was threefold higher in the BC group. The mean duration of follow-up was 5.0 years in BC with a range from 4 months to 15.3 years and 94 of the 102 hips (92%) received at least one treatment.

**Table 1:** Patient demographics of the British Columbia and India cohorts

Demographic	British Columbia	India^2^
	(*n* = 90)	(*n* = 610)
Age at presentation (95% CI), years	6.8 (6.4, 7.3)	N/A
Male	6.9 (6.4, 7.4)	9.1 (6.2, 12.0)
Female	6.5 (5.6, 7.5)	8.5 (4.9, 12.1)
Age at onset of symptoms (95% CI), years	6.2 (5.7, 6.7)	N/A
Sex, *n* (%)		
Male	70 (78)	438 (72)
Female	20 (22)	172 (28)
Affected hip, *n* (%)		
Unilateral	79 (88)	589 (96)
Left	37 (41)	N/A
Right	42 (47)	N/A
Bilateral	11 (12)	21 (4)

Sixteen percent of hips in the BC group presented as modified Waldenstrom stage Ia, 48% presented as Ib, 14% as IIa, 18% as IIb, 4% as IIIa and 1% as IIIb. In total, 97 of 102 hips (95%) presented in stages I and II, which is comparable to the 90% of children in the Indian group presenting by stage IIIa.

The modified Waldenstrom and Herring classifications demonstrated substantial agreement for both intra- and interobserver reliability (Table 2). The Salter–Thompson and Catterall classifications demonstrated slight agreement for intraobserver reliability and faired only slightly better for interobserver reliability with fair and moderate agreement respectively.

**Table 2:** Intra- and interobserver reliability of the modified Waldenstrom, Herring, Salter-Thompson and Catterall classification systems

Classification	Sample Size n*	Intraobserver		Interobserver	
		Observed Agreement	Kappa *k* (95% CI)	Observed Agreement	Kappa *k* (95% CI)
Modified Waldenstrom	100	72%	0.67 (0.59, 0.75)	80%	0.76 (0.67, 0.85)
Herring	27	89%	0.65 (0.34, 0.96)	86%	0.62 (0.27, 0.98)
Salter–Thompson	22	41%	0.07 (− 0.12, 0.27)	46%	0.24 (0.01, 0.48)
Catterall	54	48%	0.10 (− 0.02, 0.22)	74%	0.41 (0.17, 0.64)

*n number of hips that could be appropriately classified based on disease stage

**Fig. 1:**
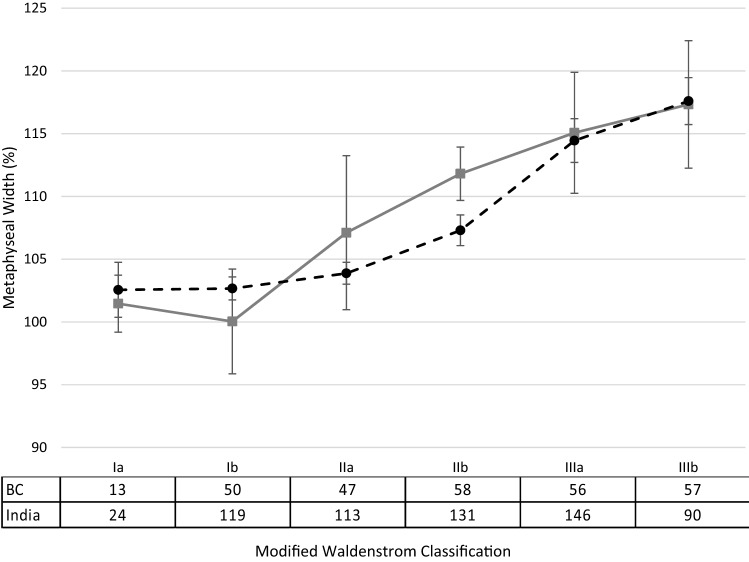
Mean percentage metaphyseal widening with 95% confidence intervals through the disease stages between British Columbia and India^2^ with sample sizes

**Fig. 2:**
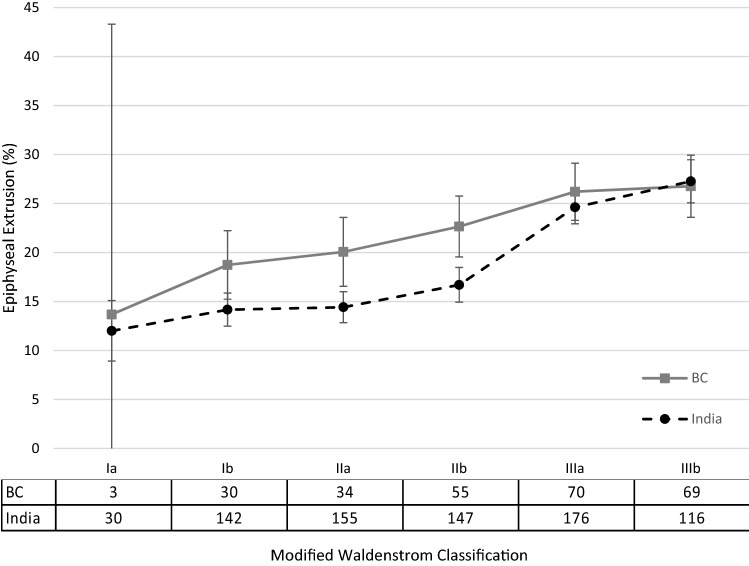
Mean percentage epiphyseal extrusion with 95% confidence intervals through the disease stages between British Columbia and India^2^ with sample sizes

In both patient groups, metaphyseal widening and epiphyseal extrusion increased as the disease progressed (Figs. 1 and 2). The greatest increase in metaphyseal widening occurred after stage Ib in the BC cohort, and after stage IIb in the Indian cohort. The final average metaphyseal widening was 119% in both groups. Mean epiphyseal extrusion exceeded 20% by stage IIa in the BC cohort, and by stage IIIa in the Indian cohort (Table 3). The final epiphyseal extrusion was similar between both groups (BC 26.8%, India 27.3%).

**Table 3:** Frequency of epiphyseal extrusion over 20%

Stage	BC		India^2^	
	Sample Size	*n* (%)	Sample Size	*n* (%)
Ia	3	1 (33)	30	6 (20)
Ib	30	11 (37)	142	41 (29)
IIa	34	17 (50)	155	55 (35)
IIb	55	31 (56)	147	66 (45)
IIIa	70	49 (70)	176	124 (70)
IIIb	69	48 (70)	116	85 (72)

The Catterall, Salter–Thompson and Herring classification systems for the BC group could be assigned in 60%, 29% and 59% of cases, respectively. The classification of the hips for both cohorts is summarized in Table 4. A high proportion of the classifiable hips were Catterall IV and Herring C in the BC cohort while comparatively fewer hips in the Indian cohort had this level of femoral head involvement.

**Table 4:** Classification of cases according to Catterall, Salter–Thompson, Herring, Mose and Stulberg in British Columbia and India

Classification system	British Columbia	India^2^
	*n* (%)	*n* (%)
Catterall system (total)	75 (60)	469 (77)
I	2 (3)	0
I I	3 (4)	30 (6)
III	10 (13)	232 (50)
IV	60 (80)	207 (44)
Salter–Thompson (total)	36 (29)	118 (19)
A	19 (53)	30 (25)
B	14 (39)	88 (75)
Herring (total)	74 (59)	360 (59)
A	0	76 (21)
B	8 (11)	130 (36)
C	66 (89)	154 (43)
Mose (total)	58 (46)	80 (13)
Spherical (good)	13 (22)	19 (24)
Flattened (gair)	20 (34)	19 (24)
Irregular (poor)	25 (43)	42 (52)
Stulberg (total)	58 (46)	N/A
Spherical congruency (I and II)*	13 (22)	N/A
Aspherical congruency (III and IV)**	45 (78)	N/A
Aspherical incongruency (V)	0	N/A

*There were no hips in class I

**There were 25 hips in class III and 20 hips in class IV

Classification of the radiographic outcome of the disease according to Mose was possible in 58 BC patients at the fully healed stage (Table 4). Irregular heads were most common followed by flattened, and then spherical heads. These findings were consistent with the Indian cohort. The final Stulberg classification for the BC group is shown in Table 4 with the majority of hips classified as aspherical.

## Discussion

The globalization of medical research is placing increased importance on understanding the natural history of disease processes in different populations. The purpose of this study was to compare LCPD between British Columbia, Canada and south-west India with respect to radiographic progression through the stages of the disease, epiphyseal extrusion and metaphyseal widening, and the final radiographic outcome. Our study found overall a similar radiographic progression of LCPD in India and BC though there were some differences identified between the cohorts. We found differences in the age at presentation, bilaterality, timing of metaphyseal widening and epiphyseal extrusion, as well as in the distribution of the Catterall, Herring, Salter-Thompson and Mose classifications between the cohorts.

There are several limitations to this study. First, the results of this study must be interpreted in the context of the retrospective study design. Challenges associated with the retrospective nature of this study include the lack of standardized follow-up between radiographs, the availability of radiographs, and the lack of Stulberg classification in the data from south-west India. Consequently, not all hips could be classified according to Catterall, Salter–Thompson and Herring classification systems. Second, we did not have an adequate number of patients to perform statistical tests and to adjust for multiple comparisons; therefore, the study is descriptive in nature. This information can be used for hypothesis-generation for future studies. We selected 2006 as our end point for inclusion due to the universal adoption of digital radiography at our institution following this year, which would make the imaging different to the study group in India. Another limitation to this study was the inclusion of non-operative treatments, such as bracing and casting, within the BC cohort. The study from south-west India did not include patients whom had undergone any form of treatment, thus comparison between the cohorts is imperfect.

Children in our series presented at a younger age compared to the Indian group. On average, both boys and girls were two years younger compared to their counterparts despite presenting at similar stages of the disease process. This trend was highlighted in recent systematic reviews of the incidence and geographic distribution of LCPD where the average age at disease onset in Europe and North America was 4.5 years compared to India and Nigeria where the average age was 9.5 and 10.3 years, respectively [10, 11].

We demonstrated substantial interobserver reliability of the modified Waldenstrom classification system comparable to that of Joseph et al. [2]. This is consistent with the validation study performed by the International Perthes Study Group which showed substantial-to-near-perfect agreement between and within observers utilizing the modified Waldenstrom classification [16]. Therefore we are confident with the disease staging in our cohort.

The patterns of epiphyseal extrusion and metaphyseal widening were similar between the two groups. An increase in epiphyseal extrusion was demonstrated after stage IIb of the disease. While we were unable to perform comparison testing, the BC cohort exceeded a mean epiphyseal extrusion of 20% earlier in the disease process than the Indian cohort (by stage IIa versus stage IIIa, respectively).

Greater epiphyseal extrusion has been associated with a poor outcome especially if it is greater than 20% [2, 3, 17, 25]. Given that a premise of surgical containment is the prevention of this migration, knowing the timing of its increase can aid in planning the timing of intervention [4, 26].

During LCPD disease, the changes in ossification of the femoral epiphysis preclude direct measurement of femoral head size from the epiphysis on plain radiographs. Metaphyseal widening has been shown to be a surrogate marker of the femoral head size and can be measured throughout the disease process [2–4] and it is predictive of coxa magna which, in turn, is associated with a poor outcome [2–4]. In the BC group, metaphyseal widening appeared to begin and have the biggest increase after phase Ib; whereas in the Indian group the greatest increase occurred after stage IIB. The earlier onset in our group may be reflective of the non-standardized radiographic follow-up in our study where the duration between radiographs may have spanned a longer period of time, and therefore, reflect slightly different points in each of the disease stages chronologically. If, in reality, metaphyseal widening increases maximally in stage IIa, it implies that femoral head flattening may be occurring early and this would warrant earlier intervention to prevent irreversible femoral head deformation.

The Mose classification defined outcome based on the shape of the femoral head as compared to perfect circular shape; with good, fair and poor descriptors attached to spherical, flattened and irregular heads respectively [21]. The number of spherical heads (good outcome) at the healed stage was similar in both groups (BC 22% and India 24%). Irregular heads (poor outcome) was the most common outcome in both the BC and Indian groups (41% and 52%), respectively. This clearly shows that less than a quarter of untreated hips in both locations do well and an unacceptably large proportion of hips have poor radiological outcomes.

Only 23% of hips in the BC group demonstrated spherical congruency (Stulberg I and II). These results are inconsistent with the findings of Herring et al. multicenter study, where 59% of children under eight years of age had a Stulberg II outcome [27, 28]. Furthermore, Wiig et al. similarly reported that in children under six years of age, Stulberg I and II hips were found in 59% of patients [9]. Our poorer radiographic outcomes are more consistent with the results of Stulberg et al. [22], who found 50% of 171 conservatively treated hips were Stulberg III or IV, and other series of older onset children such as Arkader et al. where Stulberg III and IV hips accounted for 57% in children older than nine years of age [29]. Our results could be due to the inherent poor interrater reliability of the Stulberg classification system highlighted by Neyt et al. where estimated agreement between rates was estimated at 68%, with a kappa of 0.783 [23]. Other reasons for the differences in radiographic outcomes may include differences in activity levels, weight bearing status or differences in the conservative treatment prescribed in our BC group.

## Conclusion

Our results demonstrate a similar radiographic progression of LCPD in India and North America with similar final radiographic appearances though differences in the distribution of the prognostic classification systems warrant further study. Given that LCPD affects children across the globe, it is imperative to be inclusive with prospectively collected registries. Demonstrating this similarity between these two sites suggests that results from the population in south-west India may be generalizable to North America and that their inclusion in prospective studies may be appropriate.

## Supplementary Information

Below is the link to the electronic supplementary material.

Supplementary file1 (DOCX 13 kb)
