# Supplementary material for: The Natural History of Non-operatively Managed Legg–Calvé–Perthes’ Disease
Source: Indian J Orthop. 2022 Jan 20;56(5):867–73. doi: 10.1007/s43465-021-00543-x (PMC9043051; doi:10.1007/s43465-021-00543-x)
Supplement: Supplementary file 1 — Supplementary file1 (DOCX 13 kb) [file 43465_2021_543_MOESM1_ESM.docx]

Title: The natural history of non-operatively managed Legg-Calvé-Perthes’ disease

Journal: Indian Journal of Orthopaedics

Authors: Ramez Ailabouni, Bryn O Zomar, Bronwyn L Slobogean, Emily K Schaeffer, Benjamin Joseph, Kishore Mulpuri

Corresponding Author Affiliation: BC Children’s Hospital, Vancouver, BC

Corresponding Author Email: kmulpuri@cw.bc.ca

**Online Resource 1**

**Supplemental Table 1.** The modified Waldenstrom classification system.

| **Stage** | **Description** |
| --- | --- |
| Ia | Sclerotic epiphysis without loss of height |
| Ib | Sclerotic epiphysis with loss of height but no fragmentation |
| IIa | Early Fragmentation with only one or two vertical fissures. |
| IIb | Advanced Fragmentation with no evidence of new bone |
| IIIa | Early new “porotic” bone formation lateral to the fragmented epiphysis |
| IIIb | New bone of normal texture covers more than one third of the epiphysis width |
| IV | Healing is complete |
